# Trichel pulse in various gases and the key factor for its formation

**DOI:** 10.1038/s41598-017-10118-2

**Published:** 2017-08-31

**Authors:** Yu Zhang, Qing Xia, Zhaorui Jiang, Jiting Ouyang

**Affiliations:** School of Physics, Beijing Institute of Technology, Beijing, 100081 China

## Abstract

We confirm in this paper that Trichel pulse of negative corona is a common phenomenon that can occur in various gases rather than only in electronegative gases as considered in the last 70 years since G W Trichel firstly reported. Trichel pulse is exactly a mode transition between low-current Townsend and high-current normal glow regime, two pulseless stages of negative corona with different operating conditions and ion flux threshold. The rising of the pulse corresponds to the breakdown and formation of temporal glow discharge, the decay corresponds to the destroy of temporal sheath, and the interval (or repetition frequency of pulses) is determined by the re-building of the positive ion cloud to enhance significantly the local electric field for glow discharge to form again. The positive ions play a predominant role for the pulse formation and the mode transition, while the negative ions in electronegative gas are not necessary even if they affect greatly the pulsing process.

## Introduction

Corona discharge is a self-sustained discharge which occurs in sharply non-uniform electric field, typically operated in needle-to-plate or wire-to-plate configuration. Trichel pulse has known as a famous phenomenon in negative corona for more than 70 years^1, 2^. It consists of regular impulses with short duration and changing interval depending on discharge current. There are some basic characteristics of Trichel pulses from negative corona operated in needle-to-plate or wire-to-plate configuration. They include that for a given radius of curvature of the needle (or wire), the frequency of pulses is almost independent of the inter-electrode separation and exhibits a linear dependence with the mean current intensity. The slope of that linear dependence increases with the rising pressure. The waveform of the pulsed current for a given electrode configuration and air pressure, however, is almost stable as the mean discharge current increases. The rising time of the pulses increases linearly with tip radius of cathode but inverse with air pressure. Simulations were performed to explain the pulsating corona current. Early Loeb *et al*. tried to build a theory involving successive electron avalanches, giving rise to three successors near the end of its development, to explain the pulsing phenomenon and reported their works in his publications and book^2–4^. Later Lama *et al*.^5^ did a systematic study of Trichel pulse from negative coronas in air, in combination with a theoretical analysis of the motion of the charge clouds. Aleksandrov^6^ extended Loeb’s theory by considering the parallel development of many avalanches, rather than insisting on successive avalanches, and succeeded in predicting much faster rising times for the main pulse. Morrow *et al*.^7, 8^ developed a similar theory for negative corona in oxygen that emphasizes the importance of the relatively wide plasma region which brings the electric field close to zero over much of the cathode region, and the crucial role played by attachment in converting electrons to negative ions, freezing the electric field formation in place. The above theory has been generally accepted by many researchers^9–12^. In recent years, there are still some numerical simulations performed on the evolution of negative corona discharge and the behaviors of Trichel pulses^13–17^. But these theoretical models are generally in or similar to Morrow’s frame. The simulations could reproduce the pulsed current and show the importance of negative ions (O_2_^−^, etc.) for the formation of Trichel pulse. But in most cases, the calculated waveform (shape) could not be well compared with the experimental one. Up to now, it is widely accepted that the periodic forming and clearing of the negative space charges is responsible for Trichel pulse. Actually, in most published references (e.g. refs 5–18) and almost all textbooks of gas discharge (e.g. refs 4, 19, 20), Trichel pulse is generally stated as a special phenomenon that only occurs in electronegative gases such as O_2_, SF_6_ or mixtures. “Trichel pulses are not observed in the electropositive gases N_2_ and Ar”^20^.

However, there were also some arguments on the formation mechanism of Trichel pulses. In 1988, Cernák *et al*.^21^ evidenced experimentally that the apparent similarity between the temporal current development of the initial stage of the negative point-to-plane gap breakdown in nitrogen, and the Trichel pulse current rise and its initial decay in electronegative gases. Accordingly, they suggested the two phenomena have a common mechanism and that the rapid quenching of the Trichel pulse cannot be caused solely by the negative-ion space-charge formation. Later they observed the pulsed waveforms in nitrogen even if they were noisy so that the waveform features were not easily distinguished^22^, and suggested that the negative corona (Trichel) pulse is associated with the ignition of a cathode-directed streamer in the immediate vicinity of the cathode and the subsequent formation of a glow-discharge-type cathode region at the streamer arrival to the cathode, rather than the theories by Loeb^2–4^ or Morrow^7, 8^. In 2001 Akishev *et al*.^23, 24^ reported the pulsed mode of a negative corona in nitrogen over a wide range of experimental parameters. In their systematic study, regular current pulses can be observed in the hysteresis region if the applied voltage is decreased after igniting the corona. They further proposed that the effect of positive ions near the cathode on the pulse formation should not be neglected which might distort significantly the local electric field. Moreover, the pulsating current in DC discharge is a common phenomenon under many other discharge configurations in various gases. In hollow cathode discharge (HCD) or parallel-plate discharge, the self-pulsing phenomenon has similar characteristics and may occur in almost all gases such as air, nitrogen, oxygen, argon and helium under proper conditions^25–28^. Periodic pulsating were also revealed experimentally in transversely homogeneous barrier discharges in helium at small values of the parameter *Pd* (below 500 torr · mm), which may relate to the negative differential resistance of the cathode fall region^29^. These pulsating current at constant voltage was presented to appear as a mode transition between two stable stages of a low- and a high-current discharge at least in air at atmospheric and low pressure^30, 31^. But this viewpoint did not accepted widely by most researchers due to probably the unclear image.

In this work, we present the results of Trichel pulses in non-electronegative gases, electronegative air and mixtures. The aim is to clarify the operating conditions and the true mechanism for Trichel pulse.

## Results

### Trichel pulse in argon

**Figure 1:**
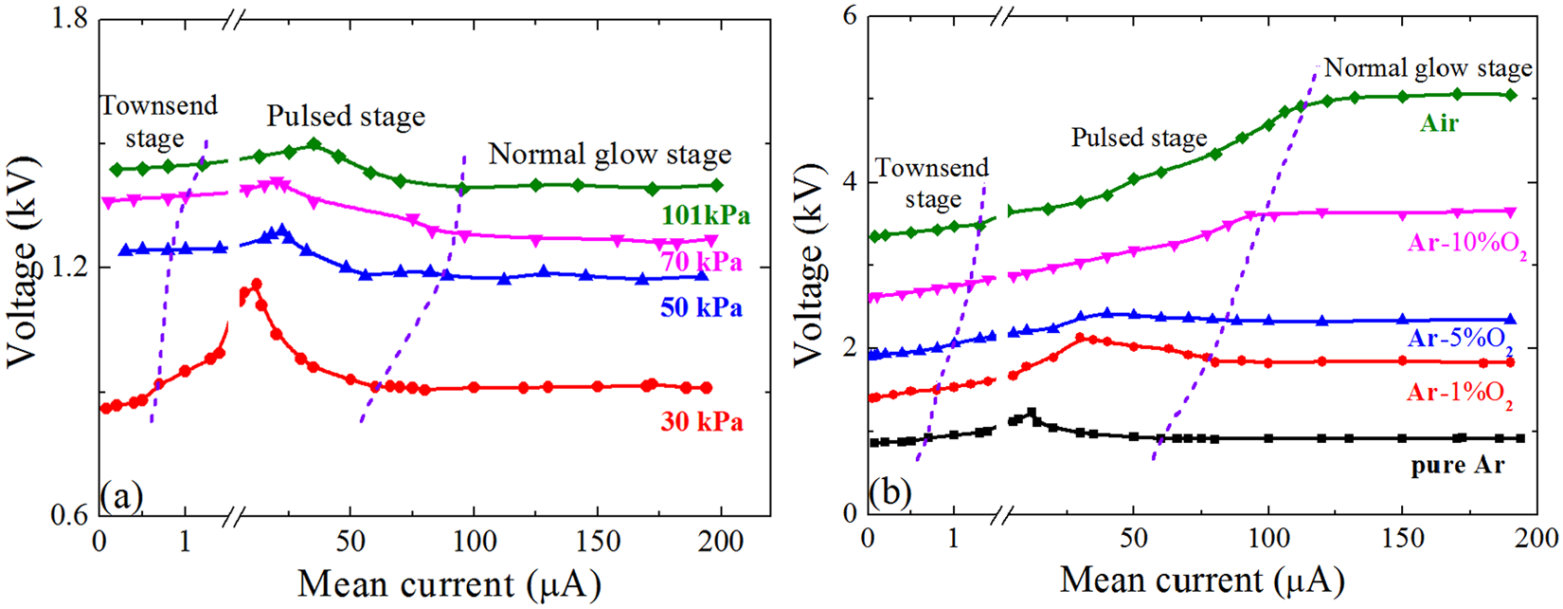
The *V-I* curves of negative coronas (**a**) in pure Ar at various pressures and (**b**) in mixtures at 30 kPa.

Firstly we operated the negative corona in pure argon (PRAXAIR, with purity of 99.999%). Trichel pulses are observed in experiments when increasing slowly the discharge current after the corona is ignited. Figure 1(a) shows the typical *V-I* curve at various pressures for tip radius *r*_0_ = 250 μm and gap spacing *d* = 5mm before spark appears.

The pulses appear between two pulseless stages of low-current (less than 1 μA, Townsend regime) and high-current (~tens μA, normal glow regime). The *V-I* curve in pulsed regime shows a positive differential resistance (PDR) firstly and then a negative differential resistance (NDR). Decreasing the gas pressure enlarges the change of voltage in pulsed regime and helps to observe more easily the self-pulsing. For example, the voltage difference rises up from 110 V at *p* = 101 kPa (atmospheric pressure) to 250 V at *p* = 30 kPa.

When some O_2_ is added into the working gas, the Trichel pulses become easier to observe. The operating voltage increases with the oxygen content and the voltage range for Trichel pulses becomes larger, as shown in Fig. 1(b) at 30 kPa. The NDR region on the *V-I* curve, however, diminishes gradually as the oxygen content increases. But if further decreasing the pressure, the NDR region can still be shown. This has also been reported by previous works^25, 26^ in low-pressure air.

### Trichel pulse in nitrogen

**Figure 2:**
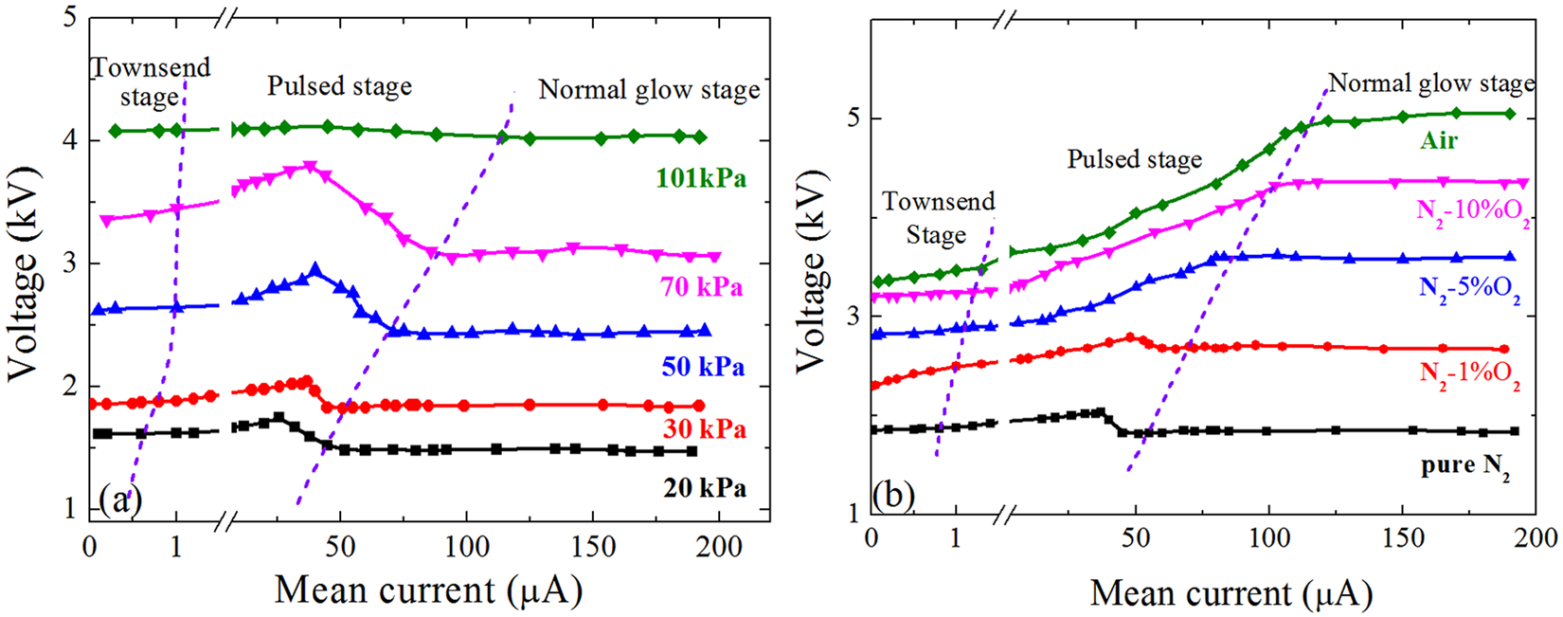
The *V-I* curves of negative coronas (**a**) in pure N_2_ at various pressures and (**b**) in mixtures at 30 kPa.

In pure nitrogen (PRAXAIR, with purity of 99.999%), Trichel pulses can also be observed. Again, the Trichel pulses appear between the low-current Townsend and the high-current normal glow discharge, as shown in Fig. 2.

However, in pure N_2_ at atmospheric pressure, the corona current is very sensitive to the applied voltage. The current increases from 1 to 115 μA when the voltage only increases from 4.08 to 4.11 kV and then decreases to 4.05 kV, resulting in appearance of 3 discharge regimes in succession. Therefore, to observe the self-pulsing, one must control very carefully the applied voltage. Or, the corona will transit suddenly from weak Townsend to strong glow discharge or vice versa. This would be the reason that the Trichel pulse was very difficult to observe in pure N_2_ in the past.

Decreasing the gas pressure helps to enlarge the voltage difference for different regimes, hence to observe much easily the self-pulsing. For instance, at 30 kPa the voltage increases from *V* = 1.87 to 2.04 kV and then decreases to 1.83 kV when the mean increases from *I*_d_ = 0.8 to 50 μA in pulsed regime. Although the voltage difference is still small, it is large enough to observe easily the stable pulses. Similar to that in pure Ar, the *V-I* curve shows both PDR and NDR in the pulsed regime, as shown in Fig. 2(a).

When some O_2_ is added into N_2_, the Trichel pulses become very easy to observe, even if the oxygen content is very small (e.g., ~1% in our experiment). The operating voltage increases with the oxygen content and the voltage range for Trichel pulses becomes larger, as shown in Fig. 2(b). The NDR region on the *V-I* curve diminishes gradually as the oxygen content increases.

### Characteristics of Trichel pulse

**Figure 3:**
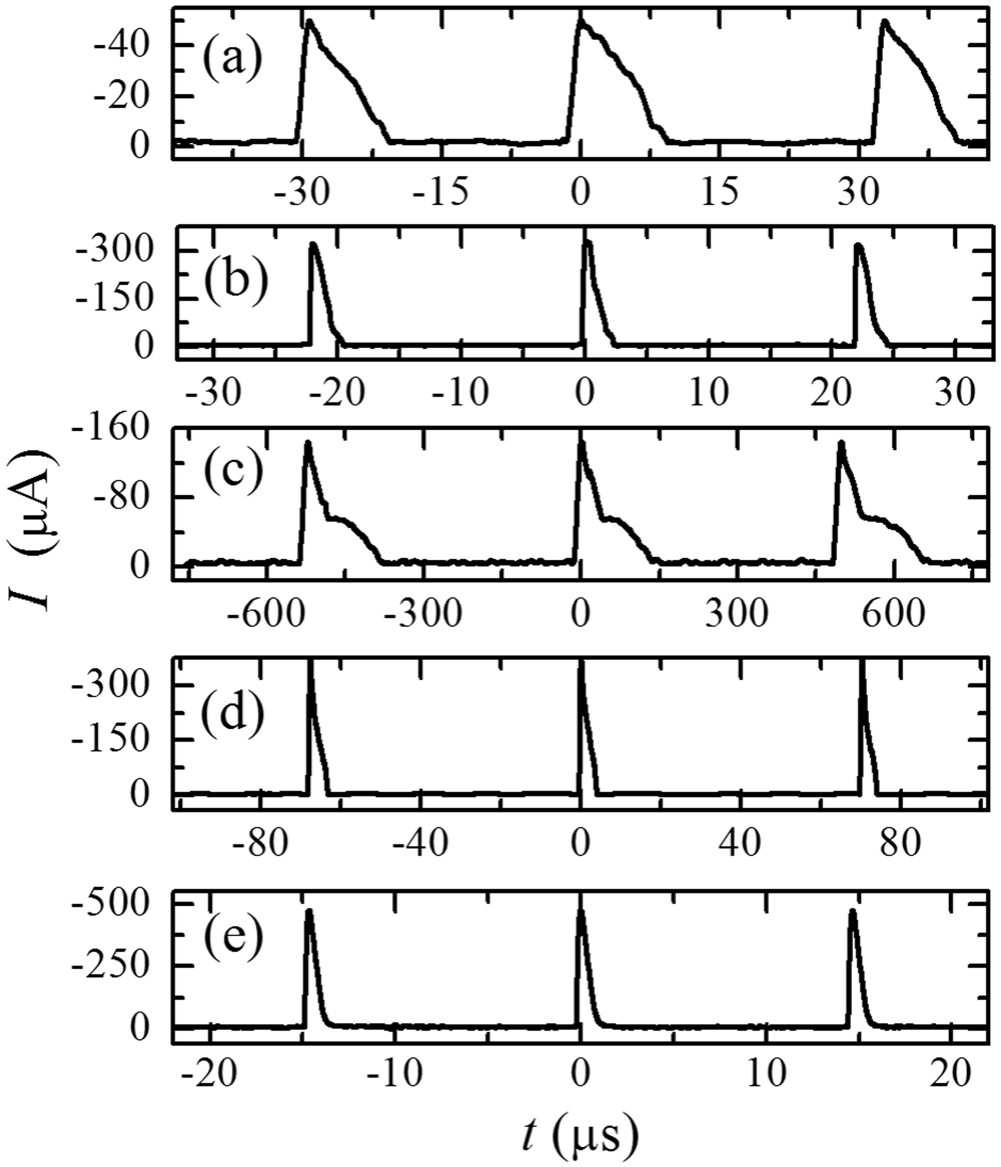
Typical waveform of Trichel pulses at *p* = 30 kPa in (**a**) pure Ar at 12 μA, (**b**) Ar-1%O_2_ at 10 μA, (**c**) pure N_2_ at 20 μA, (**d**) N_2_-1%O_2_ at 15 μA and (**e**) air at 20 μA.

The stable waveform of Trichel pulse relates only to the gas properties at given electrode geometry. Typical waveforms of the pulsed current are shown in Fig. 3 for various gases at 30 kPa.

In non-electronegative gases, the pulse is relatively weaker and broader. For example, the pulse amplitude in pure Ar or N_2_ is about 50 μA or 130 μA, much less than that in mixture of Ar-1%O_2_ (~300 μA), N_2_-1%O_2_ (~400 μA) or air (~500 μA). The rising time is much larger in Ar (~1.6 μs) or N_2_ (~18 μs) than in mixture of Ar-1%O_2_ (~0.25 μs), N_2_-1%O_2_ (~0.5 μs) or air (~0.15 μs), and the decay is longer in Ar (~9 μs) or N_2_ (~150 μs) than in mixture of Ar-1%O_2_ (~2.3 μs), N_2_-1%O_2_ (~3.8 μs) or air (~1.1 μs).

**Figure 4:**
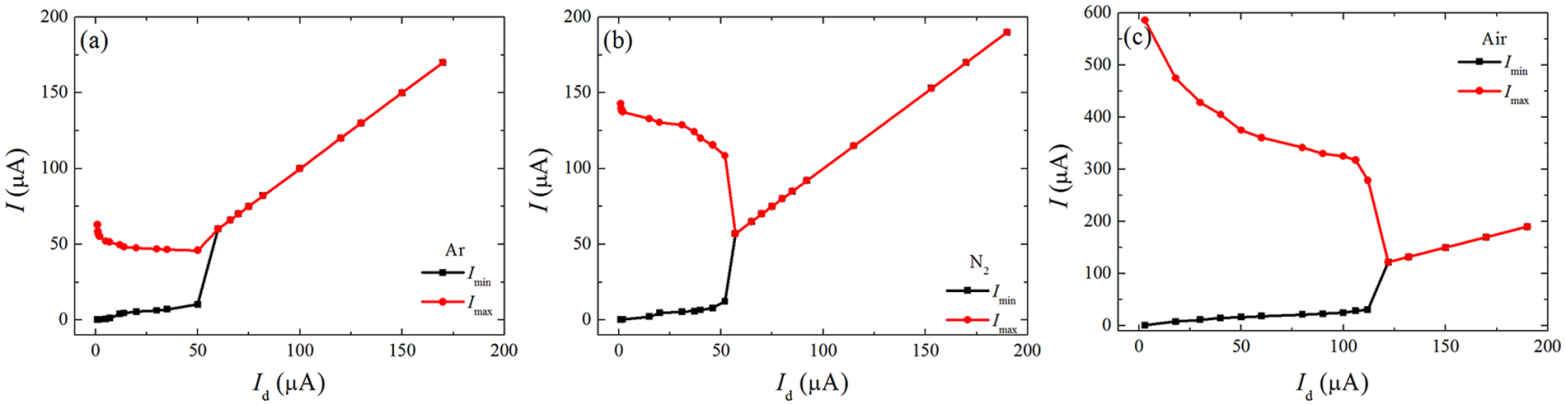
DC component and amplitude as a function of the mean current of negative corona in (**a**) pure Ar, (**b**) pure N_2_ and (**c**) air at 30 kPa.

There exists a DC component on the current during the pulse interval, as shown in Fig. 3. This DC base (or the minimum of current) is always larger than that in Townsend regime and increases slightly and proportionally with the mean current *I*_d_, as shown in Fig. 4. The DC component increases from 0.2 μA, 0.2 μA and 0.3 μA at beginning of Trichel pulse to 10.2 μA, 12.3 μA and 31 μA when the pulse disappear suddenly in argon, nitrogen and air, respectively. Meanwhile, the peak value of the current (or the maximum of current) decreases, from 62.8 μA to 46 μA in argon, 142.9 μA to 108.4 μA in nitrogen and 586 μA to 279 μA in air. Finally the minimum and maximum values become coincident when the discharge entering normal glow regime.

The other characteristics of Trichel pulses in non-electronegative gases (argon and nitrogen) follow the tendency observed in electronegative gases^30^. The pulse frequency increases linearly with the mean current, while decreasing the cathode tip radius or increasing the pressure leads to a smaller pulse rising time. It seems that the basic characteristics of Trichel pulses do not rely on the existence of electronegative components.

### Formation and decay of Trichel pulse

**Figure 5:**
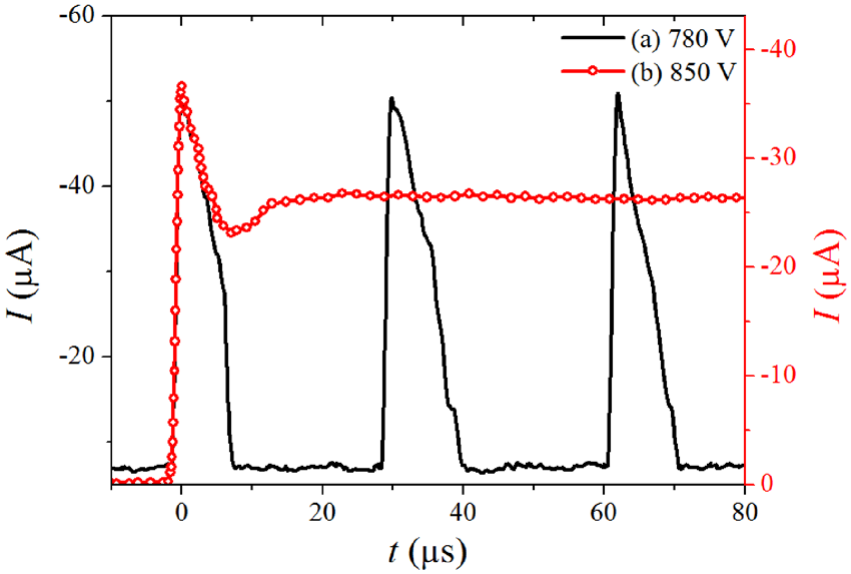
Current waveforms of (**a**) Trichel pulse at *V* = 780 V and (**b**) pulsed glow at *V* = 850 V in Ar at 30 kPa.

We then excited the corona discharge in low-pressure argon of *p* = 30 kPa by a negative pulsed voltage of fast-rising (~50 ns) at frequency of 500 Hz. In this case, there exist three kinds of discharges in each voltage pulse according to the applied voltage. That is, when the voltage is lower (less than *V* = 800 V in experiment), there appears a series of regular Trichel pulses; around a threshold of voltage (*V* = 810 V), the discharge firstly shows several Trichel pulses and then transits to stable glow; and at higher voltages (above *V* = 850 V), the discharge directly enters into normal glow after breakdown. The current waveforms at *V* = 780 V and 850 V are plotted in Fig. 5 for comparison.

It is seen that the rising edge of the pulsed current is exactly the same for Trichel pulse at *V* = 780 V and pulsed normal glow at *V* = 850 V, indicating that the formation of Trichel pulse is actually that of glow discharge. This has also been shown previously in air in ref. 30. by optical diagnostics. In experiment the Trichel pulse lasts for 9.7 μs and returns to low-current stage of *I*_d_ = 7.2 μA, while the glow discharge sustains in high-current stage of *I*_d_ = 27 μA after formation and returns to zero when the voltage pulse is off. The rising time of the both pulsed currents decreases with the increasing gas pressure.

**Figure 6:**
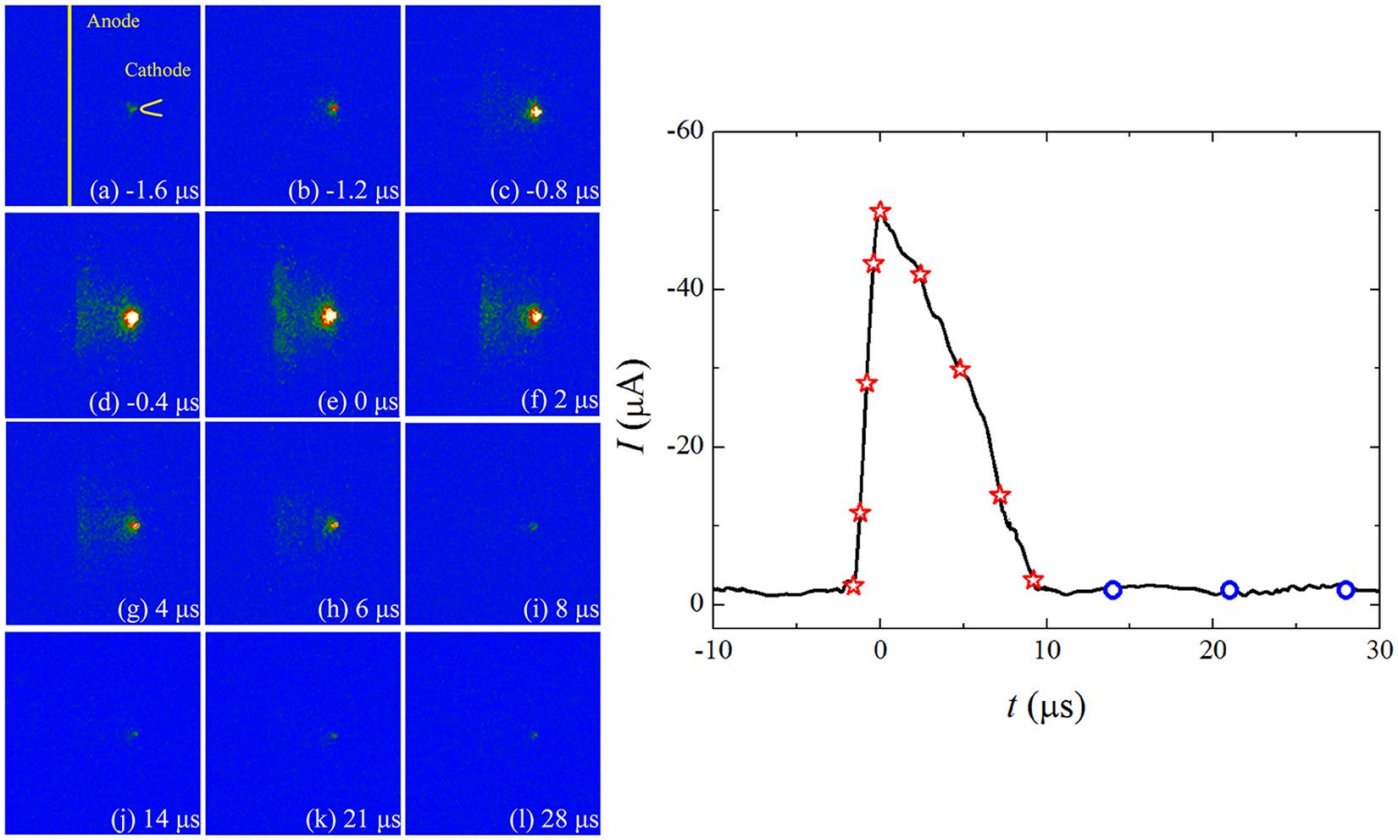
Time-resolved ICCD images of Trichel pulse in Ar at *p* = 30 kPa and *I*_d_ = 12 μA.

The spatial-temporal development of Trichel pulse in negative corona is recorded by intensified charge coupled device (ICCD) camera. Figure 6 shows the images at different times in Ar at *p* = 30 kPa and *I*_d_ = 12 μA. The ICCD images are made with gate width of 0.4 μs and accumulated in 10 cycles. The corresponding time for each frame is noted in the pulse waveform.

At the beginning of the pulse, the discharge performs as a dim dot at the tip of the cathode, as shown in Fig. 6(a). With the temporal current rising up, the discharge becomes stronger and the transition from weak Townsend mode to glow mode starts. As seen in the rising edge of the pulse in Fig. 6(b–d), the bright regions of negative glow (NG) and positive column (PC) are formed gradually. Around the pulse peak (*t* = 0 μs in Fig. 6(e)), the corona morphology performs as a typical glow pattern with the brightest NG ahead the tip, PC region spreading along the grounded electrode and Faraday dark space (FDS) between them. Thereafter, the glow-like discharge falls back to Townsend mode in the decay time. PC region disappears gradually and then NG decays to a dim dot, as shown in Fig. 6(f–i). During the pulse interval, the discharge sustains weakly in Townsend mode, as recorded under a extremely high ICCD MCP gain in Fig. 6(j–l), rather than extinguishes. The development of Trichel pulse in non-electronegative gas is exactly formation of temporal glow mode from Townsend discharge and then decays back, which is the same as that in electronegative gas (e. g. in air ref. 30).

## Discussions

As shown above, the appearance of Trichel pulses in all the non-electronegative gases and mixtures only relates to the discharge current. The features of Trichel pulses do not relate directly to the electrode separation or the applied voltage, but depend on the tip radius of cathode, the gas composition and pressure. The basic characteristics of Trichel pulses in pure Ar, N_2_ or mixtures are just the same as in air^1–5, 18–20, 30^. They include: (1) the current waveform (pulse shape) is independent on the mean current *I*_d_ at given needle radius *r*_0_ and gas pressure *p*; (2) the repetition frequency of the pulses increases linearly with *I*_d_; (3) the rising time increases with *r*_0_ but decreases with *p*; (4) the pulses always appear between two pulseless stages of Townsend and normal glow discharge. The formation of pulse corresponds to the temporal glow breakdown as evidenced by the comparison between Trichel pulse and pulsed glow. It is confirmed that Trichel pulses can really occur in non-electronegative gases like pure N_2_ and Ar, although the conditions are a little stricter than that in air or mixtures, esp. at atmospheric pressure. The only difference is the pulse shape which depends on the gas property at given electrode geometry. Decreasing pressure or adding electronegative O_2_ in Ar or N_2_ makes the Trichel pulse easier to observe. Since there is no negative ions formed in non-electronegative gases, Trichel pulse would not relate to the negative ions or their accumulation/clearance in space.

Since the low-current Townsend and high-current normal glow discharges of negative corona are two different stages, the conditions for sustaining them are distinctly different and they cannot transmit continuously from one to another. As an example, we then calculate the critical ion flux for Townsend and normal glow discharge of needle-to-plate corona in argon at atmospheric pressure.

**Figure 7:**
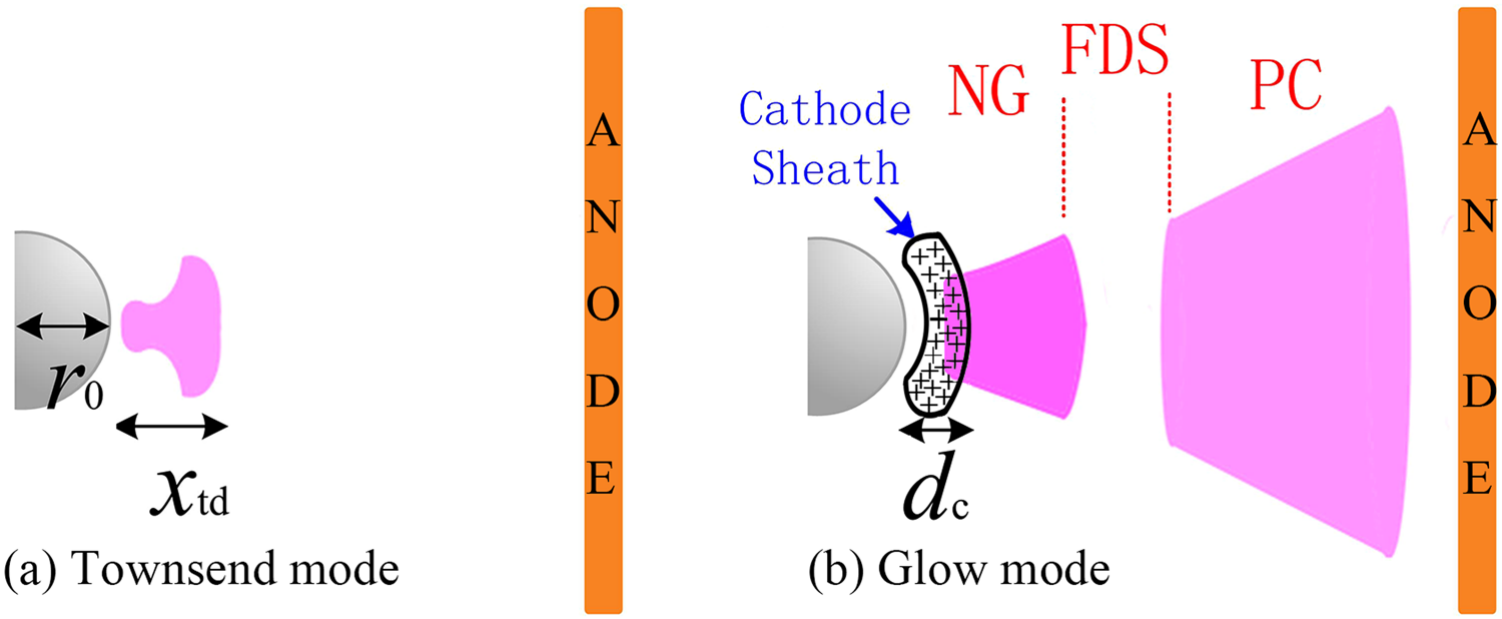
Illustration of needle-to-plate corona in (**a**) Townsend mode and (**b**) glow mode.

In Townsend regime, the corona is relatively weak and is sustained in the vicinity of cathode as described in Fig. 7(a). The space charges are not dense to distort significantly the original electric field. The electric field around the cathode tip can be estimated by parabolic tip-plane model as^20^

1 $$E(x)=\frac{2V}{({r}_{0}+2x)\,\mathrm{ln}(2d/{r}_{0}+1)},$$

where *V* is the applied voltage and *x* is the axial distance from the cathode surface. The voltage to sustain Townsend discharge is about 1.45 kV at atmospheric pressure, then the field at cathode surface is about *E*_C_ ~ 30 kV/cm. At the discharge boundary, the field falls to a level so that the ionization (or *α*-process) just cannot sustain, being about *E*_min_ = 2.5 kV/cm^20^. From Eq. (1), the active region *x*_TD_ can be calculated as:

2 $${x}_{{\rm{TD}}}=\frac{{r}_{0}{E}_{C}}{2{E}_{{\rm{\min }}}}-\frac{{r}_{0}}{2},$$

which is estimated to be *x*_TD_ ≈ 1.3 mm under the present conditions of *r*_0_ = 250 μm and *d* = 5mm. In this region, the positive ion density *n*_i_ should be no more than 10^9^ cm^−3^ in Townsend regime so that they would not accumulate in space to distort the original field significantly. Ignoring the diffusion, the maximum ion flux is *Γ*_max,TD_ = *n*_i_(*x* = 0) *μ*_i_ *E*_C_ ~ 1.8 × 10^15^ cm^−2^s^−1^ (where *μ*_i_ ≈ 2.6 cm^2^V^−1^s^−1^ at atmospheric pressure). Stable Townsend discharge can only sustain below this critical flux. Similar estimation can be done in electronegative gas, but at the boundary the ionization coefficient *α* is just equal to the attachment coefficient *η* ^19, 20^.

In normal glow regime, there exists a cathode sheath at the vicinity of the cathode tip (begins around the cathode tip and ends at the middle of the NG) as depicted in Fig. 7(b). In corona structure, the sheath shape can be treated as concentric sphere^20^ and the electric field in it is determined by the voltage drop cross sheath (or cathode fall of normal glow) *V*_n_ and sheath thickness *d*_c_

3 $$E(x)=\frac{{V}_{n}{r}_{0}({r}_{0}+{d}_{c})}{{({r}_{0}+x)}^{2}{d}_{c}}.$$

*d*_c_ and *V*_n_ are determined by air pressure and cathode material^20^, being *d*_c_ ~ 1.3 × 10^−3^cm and *V*_n_ ~ 165 V at atmospheric pressure. Since the positive ions are much denser than electrons in sheath, the field due to the ions is

4 $${E}_{i}(x)=E(x)-{E}_{0}(x)=\frac{{V}_{n}{r}_{0}({r}_{0}+{d}_{c})}{{({r}_{0}+x)}^{2}{d}_{c}}-\frac{2V}{({r}_{0}+2x)\,\mathrm{ln}(\frac{2d}{{r}_{0}}+1)}$$

At atmospheric pressure, the corona voltage for glow regime is *V* = 1.4 kV under the present conditions. The field on cathode surface is *E*_C_ ~ 124 kV/cm. From Gauss laws $\nabla \cdot E=q{n}_{+}/{\varepsilon }_{0}$, one can obtain the ion density in sheath (0 < *x* < *d*_c_) to be more than 10^12^ cm^−3^. Then the minimum ion flux for sustaining normal glow discharge is *Γ*_min,GD_ = *n*_i_(*x* = 0) *μ*_i_ *E*_C_ ≥ 3.1 × 10^17^cm^−2^s^−1^. This is at least 2-order higher than the maximum for Townsend regime (*Γ*_max,TD_ ~ 10^15^ cm^−2^s^−1^).

**Figure 8:**
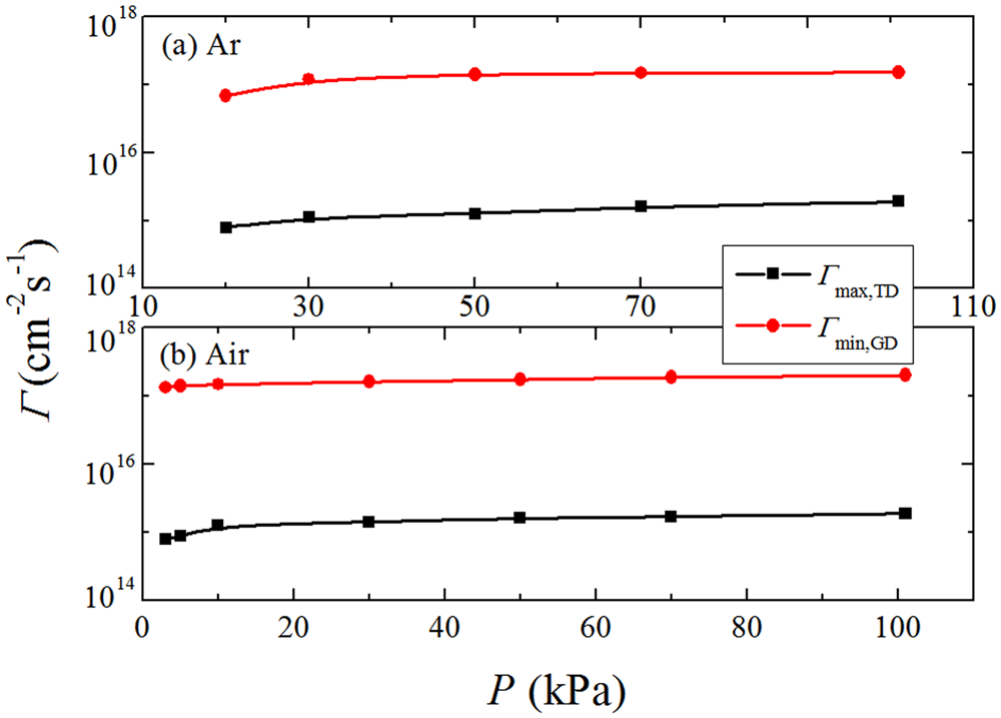
The maximal ion flux for Townsend regime and minimal ion flux for glow regime in (**a**) Ar and (**b**) Air.

In corona discharge, *Γ*_max,TD_ and *Γ*_min,GD_ are decided by gas property and electrode configuration and always follow the tendency discussed above for other gases or other pressures. Figure 8 shows the ion flux threshold in Ar or air at different pressures. The maximal ion flux for Townsend regime *Γ*_max,TD_ is always 2-order smaller and can never reach the level of the manimum for glow regime *Γ*_min,GD_.

If the ion flux (corresponding to the discharge current) is *Γ*_i_ < *Γ*_max,TD_ or *Γ*_i_ > *Γ*_min,GD_, the corona can be stabilized in pulseless Townsend or normal glow stage. If *Γ*_max,TD_ < *Γ*_i_ < *Γ*_min,GD_, the discharge will not be stable. The discharge firstly initiates in Townsend mode, but ion dissipation by drift cannot balance the generation by ionization so that ions will accumulate in space to form the positive ion cloud. Eventually, glow discharge forms when the field of space ions is stronger enough, and temporal cathode sheath forms. However, since the flux is not large enough for sustaining the sheath, the discharge cannot be stabilized in glow stage. The temporal sheath will be destroyed later due to drift, diffusion and/or recombination of space charges, and the discharge turns back to Townsend stage. Consequently, the positive ion cloud and the temporal sheath will periodically form and clear, resulting in the pulsed discharge.

From this mechanism, the pulse rising corresponds to the breakdown and formation of glow discharge, in which the positive ions play the dominant role. The rising time corresponds to the formation of temporal cathode sheath and relates to gas property and electrode geometry, increasing with cathode radius but decreasing with gas pressure, as seen in this work as well as the previous reports in air [e.g. refs 5, 18–20, 30]. The decay corresponds to destroying of the temporal sheath which is determined by dissipation of temporal cathode sheath. The interval corresponds to the re-accumulation of positive ions and hence the re-building of positive ion cloud, which is inversely to the current in Townsend stage (i.e., the DC component of the pulsed current in experiment, which is proportional to the mean current). This period is therefore related to the mean current, the gas property (composition and pressure) and the cathode radius, as shown in this work and the previous ones [e.g. refs 1–5, 18–20].

In electronegative gas, the negative ions are formed by electron attachment during the pulse process. They have some influences on the pulse characteristics. In the pulse rising, the field induced by negative ions is reversed to the applied one and offsets, to some degree, the enhancement of positive ions on the local field. Consequently, more positive ions are needed to form the temporal glow mode, which leads to larger amplitude of Trichel pulses in gases with electronegative components. In the pulse decay, the accumulation of negative ions in space is helpful to accelerate the cleaning process of positive ion cloud and hence to narrow the pulse (especially the decay time, as seen in Fig. 3).

In conclusion, Trichel pulse in negative corona discharge is a common phenomenon that can occur in various gases rather than only in electronegative gases as considered for more than 70 years. It is the mode transition from low-current Townsend to high-current normal glow discharge. The minimum ion flux (corresponding to discharge current in experiment) for normal glow discharge is much larger, at least 2-order higher, than the maximum for Townsend discharge, so that the corona discharge cannot transmit continuously from Townsend to glow regime. When the ion flux is small enough so that the ions cannot accumulate in space to distort significantly the original electric field, the discharge sustains in low-current Townsend regime. When the ion flux is large enough to sustain the cathode sheath, the discharge stably exists in high-current normal glow regime. When the flux is ranged between the two critical limits, Trichel pulses appear. The pulse rising corresponds to the breakdown and formation of temporal glow discharge, while the decay corresponds to the destroying of the temporal sheath. The pulse interval is determined by the re-building of positive ion cloud that enhances significantly the local field and insures the formation of glow discharge. The positive ions play the predominant role during the pulsing process. The negative ions formed by electron attachment in electronegative gas or mixture are dispensable, but make the pulsing process easier to happen. Sophisticated simulation is required to demonstrate the formation mechanism of negative corona (Trichel pulse) in any gas in the future.

## Methods

Experiments are performed in a typical needle-to-plate configuration similar to ref. 30. It consists of a needle cathode with tip radius of *r*_0_ = 50–250 μm and a plate anode separated by spacing of *d* = 1–20 mm. A DC high voltage is applied to the needle cathode through a ballast resistor *R*_1_ = 1 MΩ and the anode is grounded through a sampling non-inductive resistor *R*_2_ = 2 kΩ. The voltage *V* on the cathode is recorded by an oscilloscope (Tektronik DPO 4104B) through a high voltage probe (Tektronik P6015A) and the discharge current waveforms are monitored by the voltage drop across the resistor *R*_2_, or *I*_d_ = *V*_R_/*R*_2_. An Amperemeter (with precision of 1 nA) is connected in series with the anode to measure the discharge mean current, a pulsed voltage of rectangular wave is also used to compare the Trichel pulse with the pulsed glow discharge in experiment. The discharge cell is put inside a vacuum chamber so that the working gas and its pressure can be adjusted conveniently. Before the start of experiments, the chamber is evacuated to better than 10^−3^ Pa and then the working gas is admitted into the chamber. We test the negative coronas in various non-electronegative gases and mixtures at pressure of 20–101 kPa.
